# Antivenom for Neuromuscular Paralysis Resulting From Snake Envenoming

**DOI:** 10.3390/toxins9040143

**Published:** 2017-04-19

**Authors:** Anjana Silva, Wayne C. Hodgson, Geoffrey K. Isbister

**Affiliations:** 1 Monash Venom Group, Department of Pharmacology, Biomedicine Discovery Institute, Monash University, Clayton, VIC 3800, Australia; wayne.hodgson@monash.edu (W.C.H.); geoff.isbister@gmail.com (G.K.I.); 2 Faculty of Medicine and Allied Sciences, Rajarata University of Sri Lanka, Saliyapura 50008, Sri Lanka; 3 Clinical Toxicology Research Group, University of Newcastle, Callaghan, NSW 2308, Australia

**Keywords:** snake envenoming, paralysis, antivenom, neurotoxicity

## Abstract

Antivenom therapy is currently the standard practice for treating neuromuscular dysfunction in snake envenoming. We reviewed the clinical and experimental evidence-base for the efficacy and effectiveness of antivenom in snakebite neurotoxicity. The main site of snake neurotoxins is the neuromuscular junction, and the majority are either: (1) pre-synaptic neurotoxins irreversibly damaging the presynaptic terminal; or (2) post-synaptic neurotoxins that bind to the nicotinic acetylcholine receptor. Pre-clinical tests of antivenom efficacy for neurotoxicity include rodent lethality tests, which are problematic, and in vitro pharmacological tests such as nerve-muscle preparation studies, that appear to provide more clinically meaningful information. We searched MEDLINE (from 1946) and EMBASE (from 1947) until March 2017 for clinical studies. The search yielded no randomised placebo-controlled trials of antivenom for neuromuscular dysfunction. There were several randomised and non-randomised comparative trials that compared two or more doses of the same or different antivenom, and numerous cohort studies and case reports. The majority of studies available had deficiencies including poor case definition, poor study design, small sample size or no objective measures of paralysis. A number of studies demonstrated the efficacy of antivenom in human envenoming by clearing circulating venom. Studies of snakes with primarily pre-synaptic neurotoxins, such as kraits (*Bungarus* spp.) and taipans (*Oxyuranus* spp.) suggest that antivenom does not reverse established neurotoxicity, but early administration may be associated with decreased severity or prevent neurotoxicity. Small studies of snakes with mainly post-synaptic neurotoxins, including some cobra species (*Naja* spp.), provide preliminary evidence that neurotoxicity may be reversed with antivenom, but placebo controlled studies with objective outcome measures are required to confirm this.

## 1. Introduction

Snakebite is a major public health concern in the tropics. Although an accurate figure of the burden of global snakebite is unavailable, an estimate of 5.5 million annual snakebites across the globe is considered realistic [1,2]. South and Southeast Asia, sub-Saharan Africa and Latin America are the most affected regions, with more than two-thirds of the global snakebite burden reported to arise from Asia [1]. Neuromuscular paralysis due to snake envenoming is common, including envenoming by elapid snakes such as kraits (genus: *Bungarus*), cobras (genus: *Naja* and *Ophiophagus*), coral snakes (genus: *Calliophis* and *Micrurus*), taipans (genus: *Oxyuranus*), tiger snakes (genus: *Notechis*) and death adders (genus: *Acanthophis*). Snake venom induced paralysis becomes life threatening with progressive paralysis of the bulbar and respiratory muscles which requires prompt airway assistance and mechanical ventilation [3]. 

## 2. Neuromuscular Paralysis in Snake Envenoming

Snake venom neurotoxins primarily target the neuromuscular junction of skeletal muscles of which the motor nerve terminal (pre-synaptic) and the nicotinic acetylcholine receptor at the motor-end plate (post-synaptic) are the major targeted sites. This is well supported by clinical observations that neurotoxic snake envenoming almost exclusively results in flaccid paralysis [4,5,6,7,8,9,10,11,12] which is due to the blockade of neurotransmission at the neuromuscular junction by venom neurotoxins [13,14,15,16]. Neuromuscular paralysis in snake envenoming varies from mild to life threatening, depending on the degree of envenoming (i.e., quantity of injected venom reaching the circulation), the composition of the venom and potentially early therapeutic interventions. Neuromuscular paralysis is classically a descending paralysis which initially involves the eye-lid muscles, clinically manifesting as bilateral ptosis, usually within a few hours of the bite. This is followed by external ophthalmoplegia and diplopia, facial muscle weakness [4,5,7,17] with slurred speech and difficulty in mouth opening [7,18]. The paralysis then descends to the neck muscles and bulbar muscles. Involvement of bulbar muscles causes difficulty in swallowing and compromises airway protection. This is potentially life-threatening because of the risk of aspiration [19]. Neuromuscular paralysis then involves the respiratory muscles resulting in hypoventilation from decreasing tidal volumes which requires mechanical ventilation [4,9,20,21,22]. In severe neurotoxic envenoming, particularly by kraits, neuromuscular paralysis will eventually involve all limb muscles [4,23]. Recovery of neuromuscular function usually follows the reverse order of muscle involvement, with ptosis and ophthalmoplegia being the last neurological signs to disappear [4]. This unique descending sequence of muscle involvement and recovery is unlikely to be due to properties of the snake neurotoxins, but rather a combination of the size and the unique physiology of the muscles and motor nerve connections, including the redundancy of neuromuscular junctions [24,25].

Central neurological effects are rarely reported in snake envenoming, and are almost always due to indirect neurological effects of haemorrohagic or thrombotic toxicity in the central nervous system from snake venom procoagulant toxins [26,27,28,29,30]. Apparent deep coma reported after krait envenoming [23,31] suggests possible direct toxin-mediated central neurological effects. However, this may be explained as an extreme state of neuromuscular paralysis mimicking coma rather than actual coma [4,31,32,33,34]. 

## 3. Neuromuscular Junction: The Primary Target Site

The neuromuscular junction is a specialised synapse between the motor nerve and skeletal muscle fibre and serves as the final relay station in the motor pathways. The neurotransmitter, acetylcholine (ACh), is stored in vesicles within the motor nerve terminal. Depolarisation of the motor nerve terminal opens the voltage gated Ca^2+^ channels resulting an influx of Ca^2+^ into the motor nerve terminal, which triggers the fusion of vesicles to the neurolemma and release of ACh into the synaptic cleft via exocytosis. The nAChR is a pentameric transmembrane protein with a central pore which functions as a non-selective ion channel. Binding of two ACh molecules with the two agonist binding sites of the nAChR leads to a conformational change resulting in the opening ofthe central pore of the nAChR, allowing an influx of Na^+^ and depolarisation of the post-synaptic membrane. Subsequent opening of the voltage-gated Ca^+^ channels causes depolarization of the sarcolemma resulting in initiation of the muscle fibre contraction process [35,36]. Snake venom neurotoxins can disrupt the neurotransmission process at several points. Most snake neurotoxins act either on the motor nerve terminal (i.e., pre-synaptically) to prevent the release of ACh or at the nAChR on the motor-end plate (i.e., post-synaptically) by antagonising the receptor. 

Most neurotoxic snake venoms contain both pre- and post-synaptic neurotoxins, and it is common for venoms to contain several different post-synaptic toxins. However, some snakes have venoms that contain only pre-synaptic (e.g., Sri Lankan Russell’s viper, *Daboia russelii*) [37] or post-synaptic (e.g., King cobra, *Ophiophagus hannah*) toxins [38], rather than a combination of the two classes. With our rapidly expanding knowledge of snake venom proteomes and venom gland transcriptomes, many potential neurotoxins have been identified in snake venoms [38,39,40,41,42]. However, only a small proportion of these toxins have been isolated and functionally characterised.

### Neurotoxins in Snake Venoms

Pre-synaptically acting neurotoxins have been isolated from many elapids such as Kraits, Tiger snakes, Taipans and also from some viperids, and appear to be important with regard to clinically evident neurotoxicity. Snake venom presynaptic toxins belong to group I phospholipases A_2_ (elapid venoms) and group II phospholipases A_2_ (Viperid venoms) (PLA_2_), a group of Ca^2+^-dependent enzymes. The exact mechanisms by which pre-synaptic toxins enter into the motor nerve terminal and initiate their actions are unclear. Hydrolysis of phospholipids in the neurilemma of the motor nerve is believed to be the primary mode of action of the pre-synaptic PLA_2_ neurotoxins [16,43]. Based on experimental work using β-bungarotoxin, the major pre-synaptic neurotoxin in Chinese banded krait (*B. multicinctus*) venom, the key toxin-mediated event occurring in the motor nerve terminals is the necrotic degeneration of the terminal boutons [13,43,44]. In isolated nerve-muscle preparations, neuromuscular transmission failure caused by pre-synaptic toxins is usually tri-phasic. This includes an initial phase of weak inhibition of ACh release, a second prolonged phase of facilitated ACh release, and a third phase of progressive decline of neurotransmission due to necrotic degeneration of the presynaptic terminal [45]. The process of necrotic degeneration includes the depletion of synaptic vesicles through the impairment of vesicle recycling, degeneration of the mitochondria and fragmentation of the neurilemma of the nerve terminal [13,43,44,46]. Ultimately, the damaged nerve terminals withdraw from the synaptic trough resulting in empty synaptic troughs [13]. The above pathophysiological changes appear to occur for, at least, elapid PLA_2_ pre-synaptic neurotoxins [47]. The process of necrotic degeneration occurs within the first 12 h of toxin exposure. Recovery of function by re-innervation takes 3 to 5 days after exposure to the toxin, and by 7 days, re-innervation is complete [13]. Many snake venoms that are known to cause severe paralysis in humans, such as Indian krait (*B. caeruleus*) [4], Malayan krait (*B. candidus*) [6], Chinese banded krait (*B. multicinctus*) [48], coastal taipan (*O. scutellatus*) [49] and tiger snake (*N. scutatus*) [11] venoms, contain potent pre-synaptic neurotoxins (Supplementary Table S1). A detailed account of these toxins is available in the review by Harris and Scott-Davey [16].

Most snake venom toxins that act post-synaptically belong to the three finger toxin family. Three finger toxins with curare-mimetic activity are broadly categorized into two groups: i.e., short-chain α-neurotoxins that have 60–62 amino acids with four disulfide bonds and long-chain α-neurotoxins that have 66–74 amino acids with five disulfide bonds. Irrespective of their structural differences, both long- and short-chain α-neurotoxins bind to similar sites on the nAChR with high affinity [50]. Short-chain α-neurotoxins bind to the nAChR 6 to 7 times faster, and dissociate 5 to 9 times, faster than long-chain α-neurotoxins [51]. α-Neurotoxins display a variety of types of interaction with the nAChR including reversible, pseudo-irreversible and irreversible binding in neuromuscular preparations. Long-chain α-neurotoxins are known to be relatively irreversible compared to short-chain α-neurotoxins. Both agonist binding sites of the nAChR need to be occupied by ACh (or agonist) to cause conformational change and the opening of the ion channel. Therefore, even if one binding site is occupied by an α-neurotoxin there will be dysfunction of the channel. Classic examples of long-chain α-neurotoxins are α-bungarotoxin from *B. multiscinctus*, and α-cobratoxin from *N. kaouthia*. A well-known short-chain α-neurotoxin is Toxin-α from *N. nigricollis* venom. 

Recently published cobra venom proteomes (or “venomes”) suggest a high relative abundance of α-neurotoxins in Thai cobra (*N. kaouthia*) [52], King cobra (*Ophiophagus hannah*) [38] and Indian cobra (*N. naja*) [53] venoms, but negligible amounts of phospholipaseA_2_ toxins that are potentially pre-synaptic toxins. Although rare, life threatening neuromuscular paralysis has been reported in envenoming by all of the above cobra species, i.e., supporting the role of α-neurotoxins in neuromuscular paralysis in humans for at least some snakes. Detailed accounts of the structural and functional aspects of -neurotoxins are available in reviews by Barber et al. [15] and Nirthanan et al. [36]. There are other types of snake neurotoxins that are potentially important in the development of paralysis, but act at different sites compared to conventional pre-synaptic and post-synaptic toxins. Fasciculins inhibit AChE in the neuromuscular junction, allowing ACh to accumulate. These toxins have been isolated from the venoms of mamba species (*Dendroaspis* sp.). Once formed, the high affinity complex of fasciculin-AChE is very slow to dissociate [54]. Dendrotoxins, isolated from several African black mamba species, block the voltage-gated K+ channels in the nerve terminals resulting in continuous neurotransmitter release at vertebrate neuromuscular junctions. These toxins, when injected into the central nervous system, also facilitate neurotransmitter release [55]. 

## 4. Antivenoms

Antivenoms are the only antidotal treatment available for snake envenoming and have been in clinical use for over a century. Antivenoms are a mixture of polyclonal antibodies which can be whole or fractionated, F(ab)_2_ or F(ab) IgG, raised against one (i.e., monovalent) or several (i.e., polyvalent) snake venom(s) in animals such as horses, sheep, goats and donkeys [56]. Their polyclonal nature means that antivenoms consist of different antibodies against different toxin antigens in the venom. The antibody molecules bind with the toxins and (1) prevent the toxin-substrate interaction by blocking the active site, (2) form large venom-antivenom complexes preventing the distribution of the toxins from the central compartment, or (3) facilitate the elimination of toxins from the body [57,58]. Potential physico-chemical, pharmacokinetic and pharmacodynamic benefits of using monoclonal [59] and recombinant antibody [60] fragments raised against individual venom components has been experimentally explored. However, translation of such experimental antivenoms for clinical use has not yet occurred.

### 4.1. Antivenom Efficacy

The efficacy of antivenom against a particular venom is due to the ability of antivenom molecules to bind with toxins in the venom [61]. i.e., with respect to neurotoxicity, this is the ability of the antivenom molecules to bind with the neurotoxins in the venom. This is dependent on: (1) the avidity of the antivenom, which is a combined effect of the affinity constants of the different antibodies towards different toxins; (2) the relative abundance of antibodies in the antivenom against the individual neurotoxins; and (3) the relative abundance of the individual neurotoxins in the snake venom of interest. The ability of the antivenom molecules to bind with a specific venom can be quantified using an in vitro venom-antivenom binding assay, which provides useful insights into the overall ability of the antivenom to bind with the venom [62,63]. Immuno-depletion and, more recently, affinity chromatography based antivenomic approaches are useful tools in testing the ability of antivenoms to bind with specific neurotoxins or toxin groups in the venoms [64]. However, all of these approaches only demonstrate toxin binding and not neutralisation of neurotoxicity. In vitro pharmacological testing of antivenoms with chick biventer cervicis nerve-muscle preparations, frog rectus abdominis and rat phrenic nerve-hemidiaphragm preparations is useful in specifically testing antivenom efficacy towards the neurotoxic properties of the venoms [45]. Of these, the chick biventer nerve-muscle preparation is capable of differentiating post-synaptic neurotoxicity from pre-synaptic neurotoxicity [37,63,65,66]. In these experimental procedures, antivenom is first equilibrated in the organ bath that contains the tissue, and then the venom or toxin is added to the organ bath allowing the antivenom to have sufficient time to bind with the neurotoxins [45]. It therefore measures antivenom efficacy only because antivenom is present prior to venom being added. The ability of antivenoms to bind to, and prevent the neurotoxicity of both long- and short-chain post-synaptic neurotoxins as well as pre-synaptic neurotoxins has been extensively investigated in the chick biventer nerve-muscle preparation [67,68,69,70]. Some studies have also demonstrated the ability of antivenom to partially reverse neurotoxicity in the chick biventer nerve-muscle preparation [65]. This is supported by a study demonstrating the ability of antivenoms to reverse the binding of post-synaptic toxins with the nAChR [71,72]. However, neuromuscular blockade mediated by pre-synaptic toxins is not reversed by antivenom, even if the antivenom is able to bind with the toxin because of the irreversibility of pre-synaptic neurotoxicity [70]. The most widely used method for assessing antivenom efficacy is rodent lethality testing. This test calculates the dose of antivenom required to reduce the mortality of rodents by 50% defined as the median effective dose 50% (ED_50_), when the animals are given five times the median lethal dose 50% (i.e., LD_50_) of the venom. From this test the ED_50_ of antivenom is calculated, which may be useful for inter-batch comparison of antivenoms or comparison of two antivenoms [73]. However, the outcome measure of this test is the death of the animal, which could be due to any, or a combination, of the toxin classes in the venom, not necessarily an effect of neurotoxicity, or even any toxic effect relevant to humans. Discrepancy between the rodent lethality based efficacy studies with in vitro venom-antivenom binding and nerve-muscle preparation based studies has recently been demonstrated [65]. In addition, the human muscle type nAChR is relatively resistant to snake venom short-chain post-synaptic neurotoxins compared to the rodent nAChR, suggesting possible inaccuracies with the use of rodents or their tissues as models for antivenom efficacy testing for neurotoxic venoms [74]. Therefore, the rodent lethality tests as a measure of antivenom efficacy for neuromuscular paralysis in humans is highly problematic. The ability of the heterologous antivenoms to cross-neutralise the neuromuscular effects of several other snake venoms and neurotoxins has been reported in in vitro pharmacological studies [66,75,76]. The remarkable ability of Asian antivenoms to cross-neutralise Australasian neurotoxic venoms and the similar ability of Australian antivenoms to cross-neutralise Asian neurotoxic venoms was shown recently [66]. This suggests the likely presence of common antigenic regions within the post-synaptic and pre-synaptic toxins indicating the potential to develop regional or even universal neurotoxic antivenoms [66].

### 4.2. Antivenom Effectiveness

The effectiveness of an antivenom can be defined as its ability to prevent the occurrence, or reverse the effects, of venom in the clinical setting [61]. With respect to neurotoxicity, the effectiveness of the antivenom is the ability of antivenom to prevent the occurrence of neurotoxicity or to reverse established neurotoxicity in snakebite patients. It is important to consider that in addition to the efficacy of the antivenom, many other factors will contribute to the effectiveness of an antivenom. These include: (1) factors that govern the severity and the rate of development of envenoming, such as the depth of the venom injection and amount of venom injected; (2) factors related to antivenom such as the delay from the bite to antivenom administration, antivenom dose, infusion rate, the ability of the antivenom to distribute to peripheral tissues; (3) the nature and extent of neurotoxin-mediated damage such as irreversible motor nerve injury due to pre-synaptic toxins; and (4), the geographical variations of the venom composition. The variability in these factors among patients makes it difficult to achieve an objective and pure measure of effectiveness of an antivenom in the clinical setting. Therefore, the interpretation of the evidence provided in clinical studies on the antivenom for neurotoxic envenoming should be interpreted with caution.

## 5. Clinical Studies of Antivenom for Neurotoxic Snake Envenoming

Well-designed, randomized placebo-controlled clinical trials are the highest quality evidence required to demonstrate antivenom effectiveness for neurotoxicity in humans. Non-placebo controlled trials, non-randomized comparative trials and well-designed cohort studies also provide useful evidence in the absence of the former. The effectiveness of an antivenom depends on diverse factors, including the inter- and intra-species variability of venom composition of the snake. Therefore, proper case definition is also of high importance in securing the data quality of the clinical studies [77].

### 5.1. Randomised Controlled Trials

Our literature search yielded no randomized, placebo-controlled clinical trials of antivenom for neuromuscular paralysis in snake envenoming. The search identified four randomized trials and one blinded cross-over trial, in which all arms in the trials received the same or different antivenom, without any placebo controls (Table 1). 

Of the above five studies, three tested the effectiveness of an antivenom. One study compared a new monovalent antivenom with an existing polyvalent antivenom in Sri Lankan Russell’s viper (*Daboia russelii*) envenoming [78] and the second compared two doses of the new CroFab antivenom in North American Crotalid envenoming in the United States [79]. In both studies, neuromuscular paralysis was not the primary outcome, but was part of the outcome assessment. Both studies showed no difference in the outcome between the trial arms. The snake species studied in both trials primarily caused venom-induced consumptive coagulopathy or local/regional effects, and neurotoxicity is non-life threatening for both snakes [5,80]. 

A third randomised blinded trial [81] conducted in South India tested two doses of Indian polyvalent antivenom against systemic envenoming. Although this study recruited patients with neuromuscular paralysis, the outcome measures for neurotoxic envenoming were not defined and the study included a heterogenous mix of snake types with the majority being unknown/unconfirmed. The study only had four versus three patients who developed neurotoxicity in the high and low dose groups, respectively. The study concluded that a low antivenom dose is more effective than the high dose, based on the obvious finding that the low dose group received less antivenom and that the low dose group had a shorter length of stay by one day. 

The fourth randomised controlled trial [82] was a small pilot study of 15 patients with no predefined primary outcome, and no blinding of the treating doctors who were allowed to give repeat antivenom doses. In addition, the study included different types of snakes which were not balanced between groups. 

A blinded randomised controlled trial [83] tested the effectiveness of edrophonium versus antivenom, only randomising patients to three different antivenom doses with all patients receiving edrophonium. The study was very small with only eight patients, had imbalanced randomisation and the time to antivenom varied between 3 h and 96 h, with a median of 24 h. This variability makes the results difficult to interpret, particularly the difference in time to antivenom because neurotoxicity usually develops over 24 h and then begins to recover. The fact that all patient responded to edrophonium suggests that this observed effect may not be directly related to the neurotoxicity.

All of the above studies lacked controlled arms and included small numbers of patients. In addition, individual studies had critical flaws such as lack of defined primary outcomes, authentication of the snake involved, and lack of blinding of the therapeutic interventions to the observer and the patient. 

### 5.2. Non-Randomised Comparative Trials

Our search yielded three comparative trials (Table 2). One study compared the duration of neuromuscular paralysis in 27 patients with *Bungarus multicinctus* envenoming in Vietnam, treated with a new antivenom, with a historical control group of 54 patients who were not treated with antivenom [84]. The antivenom group had a shorter duration of mechanical ventilation, intensive care unit stay and other neurological signs, indicating the possible beneficial effects of antivenom in accelerating the recovery of neurotoxicity. However, there was no difference in the number of patients requiring mechanical ventilation between the two groups (>80%). This may have been due to the fact that antivenom was delayed by 19 ± 9 h. The un-blinded before and after nature of the study also meant that other changes to treatment may have occurred between the groups. Duration of ventilation and length of stay are not good objective measures and there was no pre-defined primary outcome for the study. 

A study of Thai cobra (*Naja kaouthia* and/or *N. sumatrana*) envenoming compared a group of patients who did not receive antivenom (historical) with three groups of patients who received a bolus of Thai cobra antivenom [85]. The antivenom-treated patient group had three sub-groups, each sub-group receiving a different antivenom dose (i.e., 50, 100 and 200 mL). The study showed a marked reduction in the duration of respiratory paralysis in the 100 and 200 mL groups compared to the no antivenom group. However, the study did not specify how respiratory paralysis was defined and the frequency of patients requiring ventilation was similar. The study also included a heterogenous group of patients with differing snake species and times of presentation to hospital. The study suggests that the post-synaptic neurotoxicity of Thai cobra envenoming is reversible, but a larger blinded, randomised and balanced study is required to confirm this. 

The third study retrospectively compared the recovery of the snakebite patients with neurotoxicity who were treated with high dose versus low dose antivenom [86]. The study concluded that the lower dose was as effective as the higher dose. However, this could have been a result of the loading dose of antivenom being similar in both groups, while only the maintenance dose differed. All of these studies had major deficiencies in case definition as well as the objectiveness of the outcome measures.

### 5.3. Cohort Studies

The search also yielded numerous cohort studies that discussed the effects of antivenom on neuromuscular paralysis, including studies on envenoming by elapids such as Indian krait (*Bungarus caeruleus*) [4,18,87,88], multi-banded krait (*B. multicinctus*) [48,89], Malayan krait (*B. candidus*) [48,90], common cobra (*Naja naja*) [8,21], monocellate cobra (*N. kaouthia*) [91,92,93,94], Philippine cobra (*N. philippinensis*) [7], eastern coral snake (*Micrurus fulvius fulvius*) [95], coastal taipan (*Oxyuranus scutellatus*) [96], Papuan taipan (*O. canni*) [10,97,98], tiger snake (*Notechis scutatus*) [11,99], rough-scaled snake (*Tropidechis carinatus*) [100], Papuan death adder (*Acanthophis laevis*) [101], Australian death adders (*Acanthophis* sp.) [12], Papuan black snake (*Pseudechis papuanus*) [102], and viperids such as Sri Lankan Russell’s viper (*Daboia russelii*) [5,103,104,105], Balken adder (*Viper berus bosniensis*) [17], southern tropical rattlesnake (*Crotalus durissus terrificus*) [106,107] and Mojave rattlesnake (*C. scutulatus scutulatus*) [108] and North American crotalids [109]. In addition, there was a large number of case reports on snake bite induced paralysis and the effect of antivenom. 

Almost all the above cohort studies commented on the efficacy or effectiveness of the antivenom therapy for neuromuscular dysfunction. However, apart from seven studies [4,5,11,12,97,100,103], the remaining studies had no data on serial venom antigen concentrations to enable comment on the ability of antivenom to clear free venom (efficacy). This deficiency, together with a lack of serial, well-defined objective neurological or neurophysiological parameters, makes it difficult to interpret the findings of the vast majority of the studies on the effectiveness of the antivenom in preventing or reversing paralysis, given that there was no untreated group for comparison. 

Several of the above observational studies showed that antivenom failed to reverse already established neurotoxicity, particularly in envenomings by taipans and kraits, whose venoms predominately possess pre-synaptic neurotoxins. A large cohort study of 166 Papuan taipan (*O. canni*) envenomings showed that antivenom did not reverse established neurotoxicity. In addition, neurotoxicity did not improve in any patients within 6 h, and 19% of patients developed their first signs of neurotoxicity and 37% deteriorated after antivenom [99]. A recent observational study of 33 common krait (*B. caeruleus*) envenomings in Sri Lanka found that Indian polyvalent antivenom, given as early as a median of 3.5 h after the bite, rapidly cleared circulating venom antigens from the blood. However, it did not reverse neurotoxicity and there was subsequent worsening of neuromuscular paralysis in the patients observed clinically and measured neurophysiologically [4]. These studies provide objective evidence for the poor effectiveness of antivenom in reversing the paralysis in krait and taipain envenoming, despite it being efficacious in binding free venom in krait envenoming. 

A large cohort of 245 authenticated Russell’s viper (*Daboia russelii*) bite patients from Sri Lanka found that neuromuscular paralysis continued to worsen, as evident clinically and neurophysiologically, despite Indian polyvalent antivenom clearing circulating venom antigens [5]. These observations were largely consistent with the case reports that described the failure of antivenom therapy to prevent development or worsening of paralysis in common krait [110] and Cape cobra (*Naja nivea*) [111] envenoming. 

A cohort study of 33 patients (18 envenomings) bitten by Papuan death adder (*Acanthophis* sp.) showed non-progression of the neuromuscular paralysis after antivenom therapy in 12 patients treated with death adder antivenom. In addition, a significant, rapid neurological improvement was observed in three patients [101]. In contrast, in a study of 29 definite death adder envenomings in Australia, 12 were treated with death adder antivenom and none showed a rapid improvement in paralysis despite the antivenom efficaciously clearing the venom antigens from the blood [12]. In contrast, there is evidence that early antivenom treatment decreases the frequency and severity of neurotoxic signs, and therefore prevents neurotoxicity developing in some snakes. In the study of 166 Papuan taipan envenoming, early administration of antivenom prevented intubation, with only 13.3% requiring intubation in patients given antivenom <4 h post-bite, compared to 63% in patients receiving antivenom >4 h post-bite [98]. Another study of 156 taipan envenomings also found that antivenom given within 4 h resulted in less patients requiring intubation [97]. A more recent cohort study in Australia of 40 coastal taipan (*Oxyuranus* spp.) envenomings demonstrated the efficacious binding of Australian polyvalent antivenom with the circulating venom. The same study found that early administration of antivenom was associated with a decreased frequency of neurotoxicity and requirement for intubation, indicating clinical effectiveness of the antivenom for preventing neuromuscular paralysis in taipan envenoming [96]. 

There were several case reports that reported spontaneous improvement with supportive treatment and no antivenom, in neuromuscular paralysis caused by *Naja haje* [112], *Naja kaouthia* [113], *Bungarus candidus* [114,115,116] and *Micrurus laticollaris* [117]. There were also numerous case reports that either suggested the beneficial effects of antivenom in accelerating the recovery of neuromuscular paralysis. None of these studies clearly demonstrated how antivenom therapy altered the clinical course of the paralysis, distinguishing it from the natural progression of the paralysis. Further, none of the reports provided circulating venom antigen data in-order to show the efficacious binding of the antivenom with the venom antigens.

## 6. Conclusions and Future Directions

Although antivenom use for neuromuscular paralysis in snake envenoming is established worldwide, there have been no randomized placebo-controlled clinical trials conducted on the effectiveness of antivenom therapy in preventing or reversing neuromuscular paralysis. Apart from a few studies, all the available randomized trials, comparative studies and cohort studies had deficiencies either in the case definition, study design, small sample size or in the objectivity of the measures of paralysis. A number of studies demonstrated the efficacy of antivenom in human envenoming by clearing circulating venom. Studies of snakes with primarily pre-synaptic neurotoxins (e.g., kraits, taipans) suggest that antivenom does not reverse established neurotoxicity, but early administration appears to prevent neurotoxicity or is associated with a decrease in severity for some snakes, mainly taipans. Small studies of snakes with mainly post-synaptic neurotoxins provide preliminary evidence that neurotoxicity may be reversed with antivenom, but placebo controlled studies with objective outcome measures are required to confirm this. The validity of the rodent lethality tests as pre-clinical tests of antivenom efficacy for neurotoxic envenoming in humans has been challenged and, in vitro pharmacological tests such as nerve-muscle preparation studies appear to provide more clinically meaningful information.

Future research needs to focus on well-designed studies investigating whether the early administration of antivenom will prevent neurotoxicity, both pre-synaptic and post-synaptic. This includes better case definition by using venom specific enzyme immunoassays to confirm envenoming [118], and improved objective measures of neurotoxicity, both clinical and neurophysiological [4]. In addition, there needs to be carefully designed studies investigating the ability of antivenom to reverse neurotoxicity in snakes with almost exclusively post-synaptic neurotoxins, such as cobras and the king cobra. 

## 7. Methods 

**Figure 1:**
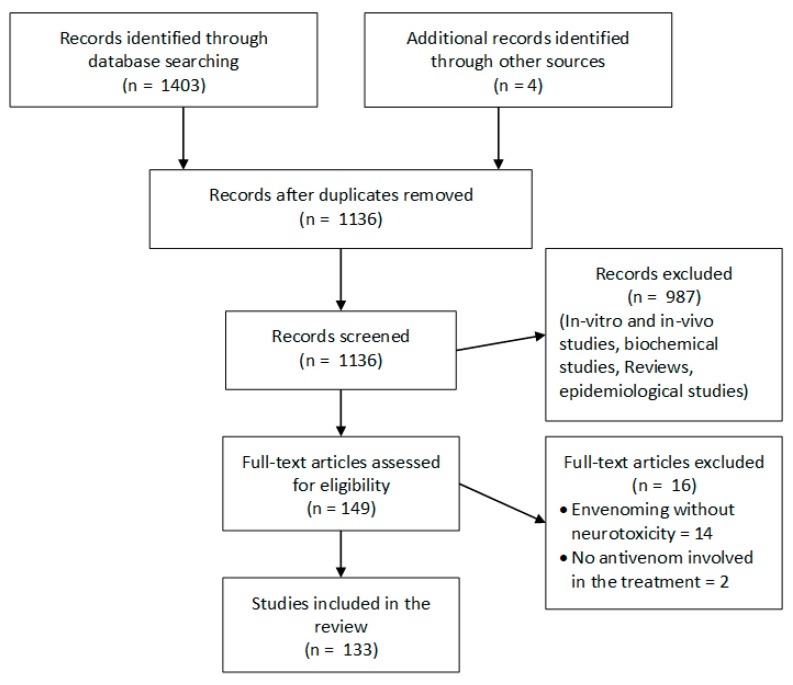
Selection of the studies for the review.

A search was carried out in MEDLINE from 1946 and EMBASE from 1947 to 31 March 2017 and included studies of snake envenoming which reported neuromuscular paralysis and provided information on antivenom. The following keywords were used: ‘‘snakebite’’, ‘‘snake envenoming/envenomation’’, “ophitoxaemia”, ‘‘neurotoxicity’’, ‘‘paralysis’’, ‘‘ptosis’’, ‘‘antivenom/antivenene’’ and ‘‘immunotherapy’’. Reference lists of retrieved articles were searched for additional relevant publications. Only articles in English were reviewed. After removing duplicates, we identified a total of 1136 studies of which 133 were included for review. (Figure 1) Articles that described in vivo and in vitro studies, reviews and epidemiological studies, clinical studies that did not report neurotoxic envenoming were excluded. There were four randomised comparative trials, one cross-over trial and 3 non-randomised comparative trials, 63 cohort studies and 62 case reports which described the effectiveness of antivenom.

## Figures and Tables

**Table 1:** Randomized trials without placebo-control and cross-over trials that describe the antivenom effectiveness for neuromuscular paralysis in snake envenoming.

Study	Number in Each Arm	Snake Species	Authentication of Snake	Trial Arms	Blinded	Randomisation	Allocation Concealment	AV ** Dose Defined	Primary Outcome Defined	Neurotoxicity Measures for Outcome	Conclusion
Ariaratnam et al. 2001	23/20	*Daboia russelii*	Identification of snake specimen and ELISA * (*n* = 43); clinical features (*n* = 3)	2 different AV (2 arms)	No	Yes	Good	Yes	Multiple outcomes	Duration of neurological signs (ptosis, diplopia)	No difference between groups
Dart et al. 2001	16/15	North American Crotalids	Not stated	2 doses of AV (2 arms)	No	Yes	Good	Yes	Yes (Improvement of a defined severity score)	Weakness Fasciculations Dizziness Paraesthesia (included to the severity score)	Both doses equally effective
Sellahewa et al.	8/7	*Naja naja* (*n* = 3) *Daboia russelli* (*n* = 11) *Hypnale hypnale* (*n* = 1)	Identification of specimen (*n* = 10), clinical features (*n* = 5)	AV vs. AV + Intravenous immunoglobulin (IVIG)	No	Yes	Good	Yes	No	Duration of neurotoxic features (ptosis and ophthalmoplegia)	No clear difference except more re-dosing in AV group.
Tariang et al. 1999	31/29	‘Cobra’, ‘viper’	Not stated	Two doses of AV	Yes	Yes	Not described	Yes	No	Not defined	Lower dose is effective than higher dose
Watt et al. 1989	2/2/4/8 (Cross-over)	Philippine cobra (*Naja philippinensis*)	ELISA (*n* = 5); Identification from pictures (*n* = 2); clinical features (*n* = 1)	Three different doses of AV and all patients received edrophonium	No	No	No	Yes	Multiple outcomes	Improvement of neurological signs	Tensilon is effective compared to AV

* ELISA: Enzyme-Linked Immunosorbent Assay; ** AV: antivenom.

**Table 2:** Comparative clinical studies that describe the antivenom effectiveness for neuromuscular paralysis in snake envenoming.

Study	Number in Each Group	Snake Species	Authentication of Snake	Study Groups	Antivenom Dose Defined	Neurotoxicity Measures	Conclusion	Remarks
Agarwal et al. 2005	28/27	Not defined	Not defined.	High vs. low dose AV	Yes	Duration of the mechanical ventilation, duration of ICU stay	No difference in outcome between the two groups	Same AV loading dose was given to both groups. The difference was only the maintenance dose.
Hung et al. 2010	27/54	*Bungarus multicinctus*	Not defined	AV vs. no AV	Yes	Number of patients requiring mechanical ventilation, duration of mechanical ventilation, length of stay in the ICU, duration of a defined degree of muscle paralysis	AV group had shorter duration of ventilation, ICU stay and other neurological signs. No difference between the number of patients requiring ventilation.	The no AV group is a historical group; AV dose varied within the AV group; Bite-to-AV delay is 19 ± 9 h (range: 5–38 h).
Pochanugool et al. 1997	27/41	*Naja kaouthia Naja sumatrana*	Patient’s description of snake or physician’s identification of the snake	No AV vs. AV (three unbalanced dose groups within AV group)	Yes	Duration of respiratory failure	Two dose groups of antivenom (100 and 200 mL) had significantly lower duration of respiratory failure compared to no AV	The no AV group is a historical group; No definition of ‘respiratory paralysis’.

## Supplementary Materials

The following are available online at www.mdpi.com/2072-6651/9/4/143/s1, Table S1: Some pre-synaptic toxins isolated and pharmacologically characterised from snake venoms that cause paralysis in humans.
