# Supplementary material for: Antivenom for Neuromuscular Paralysis Resulting From Snake Envenoming
Source: Toxins (Basel). 2017 Apr 19;9(4):143. doi: 10.3390/toxins9040143 (PMC5408217; doi:10.3390/toxins9040143)
Supplement: Supplementary file 1 [file toxins-09-00143-s001.pdf]

Supplementary Materials: Antivenom for Neuromuscular Paralysis Resulting From Snake Envenoming

Anjana Silva, Wayne C. Hodgson and Geoffrey K. Isbister

Table S1. Some pre-synaptic toxins isolated and pharmacologically characterised from snake venoms that cause paralysis in humans.

Snake	Toxin	Structure	M.W. (Da)	Relative Abundance in the Venom	Pharmacology
Bungarus multicinctus	β-bungarotoxin	Heterodimer (Chain A: with PLA_2 activity; chain B: polypeptide homologus to Kunitz-type proteinase inhibitor)	21,800	40.75% in specimens from Vietnam [1]	Potent pre-synaptic neurotoxicity; no myotoxic activity.
Oxyuranus scutellatus	Taipoxin	Trimer (1:1:1 complex of three PLA_2 isoforms, α, β and γ-taipoxin)	47,600	20.4% [2]	Potent pre-synaptic neurotoxicity and myotoxicity.
Notechis scutatus	Notexin	Monomer (has Ns and Np isoforms)	13,600	unavailable	Potent pre-synaptic neurotoxicity and myotoxicity.
Oxyuranus canni	Cannitoxin	Trimer (1:1:1 complex of three PLA_2 isoforms, α, β and γ subunits.	44,848	16% [3]	Potent pre-synaptic neurotoxicity and myotoxicity.
Crotalus durissus terrificus	Crotoxin	Non-covalently linked Crotoxin A (catalytically inactive) and B (catalytically active). Several isoforms of subunits are present.	23,400	~50% [4]	Moderate pre-synaptic neurotoxicity and myotoxicity
Daboia russelii	U1-viperitoxin-Dr1a	Monomer	13,641	19.2% [5]	Weak Pre-synaptic neurotoxicity and weak myotoxicity.
Oxyuranus microlepidotus	Paradoxin	Trimer (1:1:1 complex of three PLA_2 isoforms)	~46,000	unavailable	Strong pre-synaptic neurotxicity
Acanthophis praelongus	P-EPTX-Ap1a	Hetero trimer	40,719	unavailable	Pre-synaptic neurotoxicity
Acanthophis rugosus	P-EPTX-Ar1a	Hetero trimer	40,879	6% [6]	Pre-synaptic neurotoxicity, myotoxicity

References

1. Ziganshin, R.H.; Kovalchuk, S.I.; Arapidi, G.P.; Starkov, V.G.; Hoang, A.N.; Thi Nguyen, T.T.; Nguyen, K.C.; Shoibonov, B.B.; Tsetlin, V.I.; Utkin, Y.N. Quantitative proteomic analysis of Vietnamese krait venoms: Neurotoxins are the major components in Bungarus multicinctus and phospholipases A2 in Bungarus fasciatus. *Toxicon* **2015**, *107*, 197–209.
2. Barber, C.M.; Isbister, G.K.; Hodgson, W.C. Solving the "Brown snake paradox": in vitro characterisation of Australasian snake presynaptic neurotoxin activity. *Toxicol. Lett.* **2012**, *210*, 318–323.
3. Kuruppu, S.; Reeve, S.; Banerjee, Y.; Kini, R.M.; Smith, A.I.; Hodgson, W.C. Isolation and pharmacological characterization of cannitoxin , a presynaptic neurotoxin from the venom of the Papuan Taipan (*Oxyuranus scutellatus canni*). *J. Pharmacol. Exp. Ther.* **2005**, *315*, 1196–1202.
4. Sampaio, S.C.; Hyslop, S.; Fontes, M.R.M.; Prado-Franceschi, J.; Zambelli, V.O.; Magro, A.J.; Brigatte, P.; Gutierrez, V.P.; Cury, Y. Crotoxin: Novel activities for a classic beta-neurotoxin. *Toxicon* **2010**, *55*, 1045–1060.
5. Silva, A.; Kuruppu, S.; Othman, I.; Goode, R.J.A.; Hodgson, W.C.; Isbister, G.K. Neurotoxicity in Sri Lankan Russell's viper (*Daboia russelii*) envenoming is primarily due to U1-viperitoxin-Dr1a, a Pre-Synaptic neurotoxin. *Neurotox. Res.* **2016**, *31*, 11–19.
6. Chaisakul, J.; Parkington, H.C.; Isbister, G.K.; Konstantakopoulos, N.; Hodgson, W.C. Differential myotoxic and cytotoxic activities of pre-synaptic neurotoxins from papuan taipan (*Oxyuranus scutellatus*) and irian jayan death adder (*Acanthophis rugosus*) venoms. *Basic Clin. Pharmacol. Toxicol.* **2013**, *112*, 325–334.
